# Hydroxyapatite Mineralization on the Calcium Chloride Blended Polyurethane Nanofiber via Biomimetic Method

**DOI:** 10.1007/s11671-010-9737-4

**Published:** 2010-08-19

**Authors:** R Nirmala, Ki Taek Nam, R Navamathavan, Soo-Jin Park, Hak Yong Kim

**Affiliations:** 1 Bio-Nano System Engineering, College of Engineering, Chonbuk National University, Jeonju 561 756, South Korea; 2 Center for Healthcare Technology and Development, Chonbuk National University, Jeonju 561 756, South Korea; 3 School of Advanced Materials Engineering, Chonbuk National University, Jeonju 561 756, South Korea

**Keywords:** Polyurethane, Electrospinning, Nanofibers, Simulated body fluid, Bioactivity

## Abstract

Polyurethane nanofibers containing calcium chloride (CaCl_2_) were prepared via an electrospinning technique for the biomedical applications. Polyurethane nanofibers with different concentration of CaCl_2_ were electrospun, and their bioactivity evaluation was conducted by incubating in biomimetic simulated body fluid (SBF) solution. The morphology, structure and thermal properties of the polyurethane/CaCl_2_ composite nanofibers were characterized by means of scanning electron microscopy (SEM), field-emission scanning electron microscopy, energy dispersive X-ray spectroscopy, X-ray diffraction, Fourier transform infrared spectroscopy and thermogravimetry. SEM images revealed that the CaCl_2_ salt incorporated homogeneously to form well-oriented nanofibers with smooth surface and uniform diameters along their lengths. The SBF incubation test confirmed the formation of apatite-like materials, exhibiting enhanced bioactive behavior of the polyurethane/CaCl_2_ composite nanofibers. This study demonstrated that the electrospun polyurethane containing CaCl_2_ composite nanofibers enhanced the in vitro bioactivity and supports the growth of apatite-like materials.

## Introduction

Electrospinning of biologically significant polymers has increased dramatically since the electrospun membranes were identified as a candidate for guided tissue regeneration applications [1-4]. In particular, polyurethane is a thermoplastic, biodegradable, biocompatible polymer with excellent mechanical and physical properties [5,6]. Due to these merits, polyurethane nanofibers have been found to be a very promising material of interest. It has been already reported that the electrospun membranes have the potential to promote osteoblastic cell function and bone regeneration [7]. Recently, the feasibility of incorporating non-electrospinnable inorganic nanoparticles into polymer solution to form composite nanofibers has attained electrospinning as an attractive technique in meeting some specific functional applications [8-12]. There were some reports on electrospun composite nanofibers, such as poly (ε-caprolactone)/tricalcium phosphate [1], hydroxyapatite/gelatin [3], silk/hydroxyapatite [13], poly-lactic acid/hydroxyapatite [14], poly (vinyl alcohol) coated poly (ε-caprolactone) [15] and triphasic hydroxyapatite/collagen/poly(ε-caprolactone) [10,11] had been analyzed and explored for potential bone regeneration applications. However, the effect of calcium chloride (CaCl_2_) on the bioactivity analysis in polyurethane nanofibers has not been characterized so far. It is interesting to note that the use of CaCl_2_ salt is salient to entrap the cells and make them grow faster [16]. Calcium facilitates the attachment of cells to scaffold and to one another. Calcium is widely used in all classic media, which is utilized in biomanufacturing and in the tissue culture applications. The bioactivity of these materials can be attributed to the formation of a biologically active bone-like carbonate containing apatite layer. Therefore, we have employed for the first time CaCl_2_ incorporation in the polyurethane nanofibers during electrospinning to enhance the bioactivity behavior of the composites.

In this study, in order to obtain the role of CaCl_2_, we synthesized polyurethane nanofiber mats with different concentrations of CaCl_2_ salt. The hydroxyapatite was coated by the biomimetic method on as-spun nanofibers by incubating in the simulated body fluid (SBF) solution for different timings to observe the formation of apatite-like materials. We performed extensive analysis to confirm the formation of apatite-like materials on the electrospun polyurethane/CaCl_2_ composite nanofibers, and we proposed a likely process for the mineralization of hydroxyapatite after incubating in the SBF solution.

## Experimental Details

Polyurethane (MW = 110,000) was purchased from Cardio Tech, Japan. 2-butanone (MEK) and NN-dimethylformamide (DMF) (analytical grade, Showa, Japan) were used as solvents without further purification. Polyurethane solution with 10 wt% was prepared by dissolving in MEK and DMF. Polyurethane with 0, 1, 1.5 and 2 wt% of CaCl_2_ (Sigma-Aldrich, USA) was used to prepare nanofiber mats. The polymer solution with CaCl_2_ was kept for 12 h to dissolve completely in the solvent. Adding more amount of CaCl_2_ in the solution did not dissolve properly, which resulted in the non-electrospinnability of the polymer solution. Therefore, we optimized the CaCl_2_ content (up to 2 wt%) in the polymer solution to obtain a complete mixing. A high voltage power supply (CPS-60 K02V1, Chungpa EMT, South Korea) of 22 kV to the syringe micro-tip was supplied to electrospin the nanofibers. The polymer solution was fed to the 5-ml syringe with a plastic micro-tip. The tip-to-collector distance was kept at 15 cm. During electrospinning, the drum was rotated at a constant speed by a DC motor to collect the developing nanofibers. All experiments were performed at room temperature.

The in vitro bioactivity analysis was performed by using SBF solution. The recipe for the preparation of SBF was adopted from the method reported elsewhere [17]. The SBF solution used in the present study was five times the concentration than that of calcium and phosphate ions when compared to conventional SBF [18,19]. The morphology of the as-spun and immersed polyurethane/CaCl_2_ composite nanofibers in SBF solution was observed by using scanning electron microscopy (SEM, S-7400, Hitachi, Japan). The SEM images were obtained for different durations (2 h, 4, 6 and 7 days) of incubation in SBF to monitor the mineralization of apatite-like materials on the nanofiber surfaces. The chemical composition of the polyurethane/CaCl_2_ composite nanofibers was analyzed by using energy dispersive X-ray (EDX) spectrometer attached to the field-emission scanning electron microscope (FE-SEM). Structural characterization was carried out by X-ray diffraction in a Rigaku X-ray diffractometer (XRD) operated with Cu-Kα radiation (*λ* = 1.540 Å). The bonding configurations of the samples were characterized by means of Fourier transform infrared (FTIR) spectroscopy. Thermogravimetric analysis (TGA, Perkin-Elmer, USA) was carried out for the electrospun mats under nitrogen ambient with a flow rate of 20 ml/min. The samples were heated from 30 to 600°C at a rate of 10°C/min, and the differential thermogravimetry graph was recorded.

## Results and Discussion

**Figure 1:**
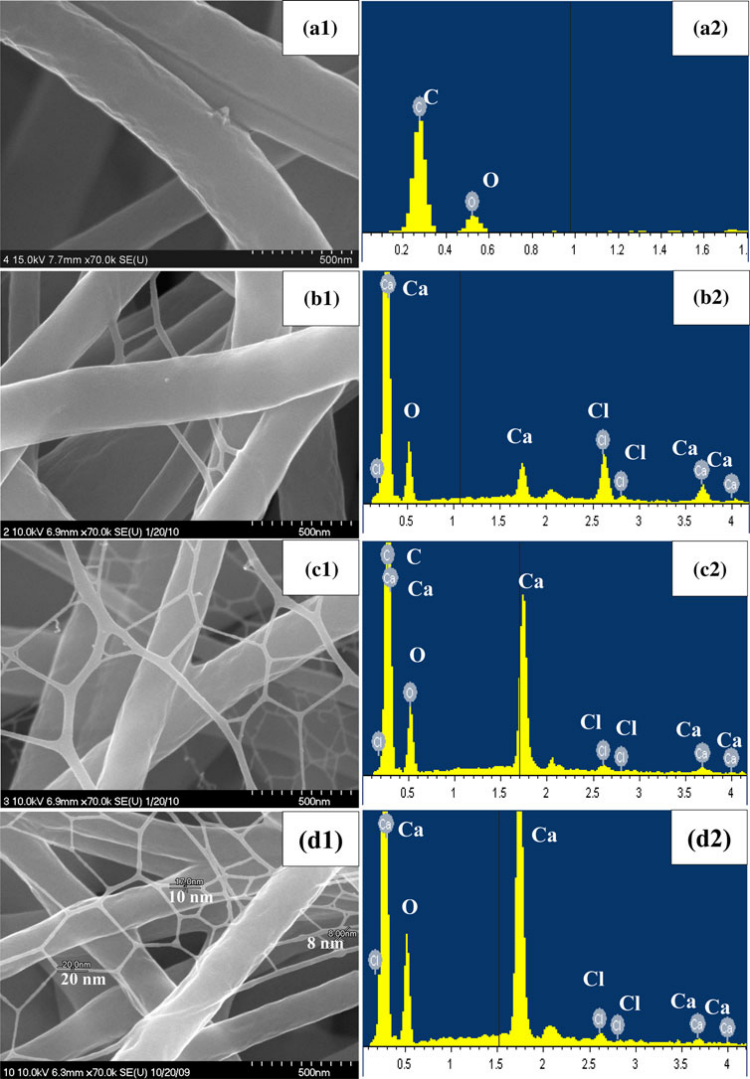
**High magnification FE-SEM images of electrospun polyurethane nanofibers containing different concentration of CaCl_2_ salt before incubation in SBF solution (a1) 0, (b1) 1, (c1) 1.5 and (d1) 2 wt% and the corresponding EDX spectra (a2)–(d2), respectively**.

The CaCl_2_ salt was mixed in the polyurethane polymer solution and then fabricated as composite nanofibers. Figure 1 shows the FE-SEM images and EDX spectra of the electrospun polyurethane nanofibers containing different concentration of CaCl_2_ salt with 0, 1, 1.5 and 2 wt%. These as-spun polyurethane nanofibers exhibited a smooth surface and uniform diameters along their lengths. By varying the experimental parameters such as, applied voltage, syringe micro-tip distance, solvent concentration, amount of salt content and flow rate, one can obtain highly uniform ultrafine nanofibers with different pore sizes. As shown in Figures 1b12d1, mesh-like ultrafine nanofibers were formed when we added the CaCl_2_ salt in the polymer solution. This kind of phenomenon is in good agreement with those of our previous reports [21], which can be utilized for the biomedical applications as the bone regenerative materials. As expected, the addition of CaCl_2_ can decrease the fiber diameter and also forming ultrafine mesh–shaped nanofibers in between the main fibers. The density of these mesh-like nanofibers was increased monotonically with increasing CaCl_2_ concentration from 1 to 2 wt%. The diameter of these ultrafine nanofibers (~8–20 nm) was of one order lower than those of the main fibers (~200–400 nm), which resulted in a large surface area-to-volume ratio and interconnected porosity. These features are considered to be of particular interest as they mimic the extracellular matrix as present in the natural tissue and have been shown to induce a significant increase in protein absorption and cell adhesion, compared to their micro-size counterparts [20]. In this study, in order to investigate the effect of CaCl_2_ on the formation of apatite nucleation in the SBF solution, different concentrations of CaCl_2_ incorporated in the polyurethane nanofibers were synthesized.

**Figure 2:**
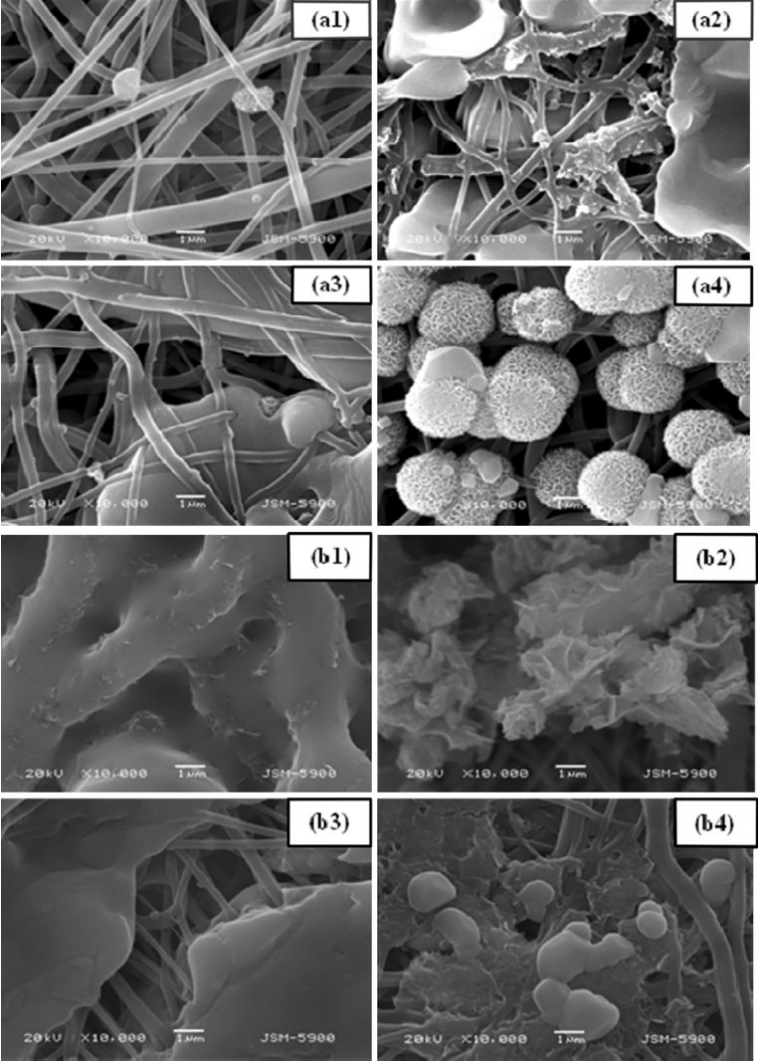
**SEM images of electrospun polyurethane nanofibers containing different concentration of CaCl_2_ after 2 h (a) and 7 days (b) incubated in SBF solution for (1) 0, (2) 1, (3) 1.5 and (4) 2 wt%, respectively**.

The chemical composition of the as-spun nanofiber mats was analyzed by EDX spectrometer attached with FE-SEM. Figure 1a2–d2 show the EDX spectra of the polyurethane nanofibers containing different concentration of CaCl_2_ salt with 0, 1, 1.5 and 2 wt%. As shown in Figure 1a2, the signals of carbon and oxygen were only observed for pristine polyurethane nanofibers. The EDX spectra revealed the presence of Ca and Cl in polyurethane nanofibers containing CaCl_2_ salt. By adding CaCl_2_ salt in polyurethane nanofibers, their compositions were increased dramatically with increasing concentration as shown in Figure 1b2–d2. The successful blending of CaCl_2_ salt with polyurethane nanofibers was confirmed by the EDX spectra.

**Figure 3:**
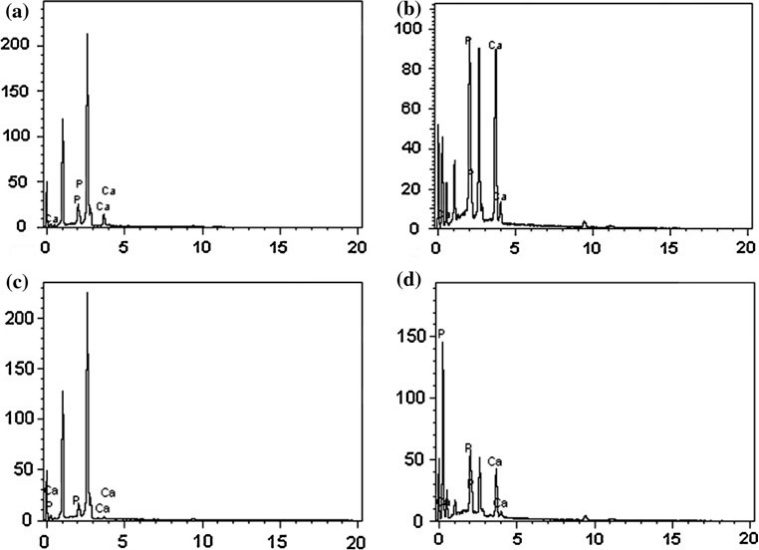
**SEM-EDX spectra of electrospun polyurethane nanofibers containing different concentration of CaCl_2_ salt after 7-day incubation in SBF solution (a) 0, (b) 1, (c) 1.5 and (d) 2 wt%**.

To observe the role of CaCl_2_ incorporation in polyurethane nanofibers for the formation of apatite-like materials after the incubation in SBF solutions, SEM analysis was performed. Figure 2a, b show the SEM images of polyurethane nanofibers containing different concentration of CaCl_2_ salt with 0, 1, 1.5 and 2 wt% after immersion in SBF solution for 2 h and 7 days, respectively. As expected, the pristine polyurethane nanofibers did not show any formation of apatite-like materials as shown in Figure 2a1. However, the formation of apatite-like materials was gradually commenced, and their size was increased monotonically with increasing CaCl_2_ concentration. As shown in Figure 2a2–a4, plenty of apatite-like materials nucleated after 2-h immersion in SBF solution. Figure 2b shows the SEM images taken after 7 days, which revealed the intense formation of apatite-like materials throughout the polymer mat. At the same time, as the incubation duration increased the formation of apatite-like materials was increased monotonically. Therefore, at the same interval, more apatite was deposited on CaCl_2_ incorporated polyurethane nanofibers than on the pristine one. Generally, the apatite-like material formation does not occur spontaneously on most of the synthetic polymers without pretreatment to activate their surfaces [22,23]. In such case, appropriate surface modification should be required to activate the nanofiber surfaces to enhance the nucleation and adhesion of apatite-like materials. However, polyurethane is a biomaterial in which the CaCl_2_ can be incorporated easily by electrospinning process and revealed excellent ability of apatite formation on its surface after soaking in SBF solution. The formation of apatite-like materials can be attributed to the partial dissolution of CaCl_2_ salt and the subsequent release of calcium ion. The exposure of calcium on the polyurethane nanofibers surfaces can provide nucleation sites for apatite-like material formation and favors further enhanced growth. To confirm the formation of apatite-like materials in the polymer mats after immersion in SBF solution, we performed the SEM-EDX analysis. Figure 3 shows the SEM-EDX spectra of polyurethane nanofibers containing different concentration of CaCl_2_ salt after incubation in SBF solution for 7 days. EDX data clearly confirmed the presence of Ca and P in the polymer mat.

**Figure 4:**
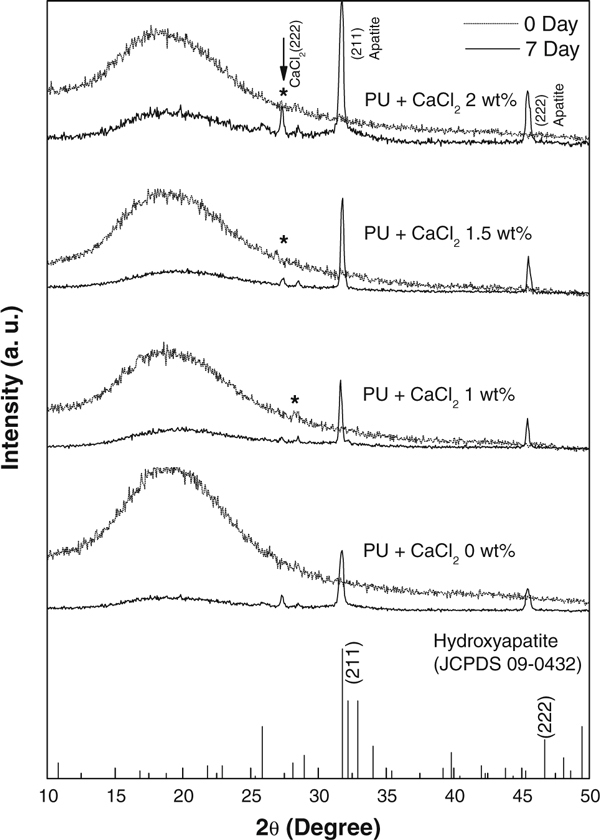
**XRD patterns of electrospun polyurethane nanofibers containing different concentration of CaCl_2_ salt before and after incubation in SBF solution**.

The X-ray diffraction pattern was used to identify the crystallographic structure of the polyurethane/CaCl_2_ composite nanofibers before and after incubation in SBF solution. Figure 4 shows the XRD patterns of polyurethane nanofibers with different CaCl_2_ concentrations before and after soaking in SBF solution. A broad peak at about 2θ = 19° was exhibited in all the samples, which correspond to the characteristic diffraction pattern for the polyurethane polymer. This diffraction peak appears to be very broad due to the amorphous nature of polyurethane. Besides, a feeble peak, which corresponds to the (222) CaCl_2_ plane, was observed at 24.5°. The diffraction patterns of all samples after 7-day incubation in SBF solution showed two clear peaks at 31.8 and 46.5° corresponding to (211) and (222) main reflection planes, which coincided well with the standard data for hydroxyapatite (JCPDS No. 09-0432). The XRD data clearly confirmed that the apatite-like materials were also formed on pristine polyurethane nanofibers. However, the intensity of the peak was increased with increasing CaCl_2_ concentration. The diffraction peaks of polyurethane/CaCl_2_ composite nanofibers after incubation in SBF were observed to be sharper and stronger suggesting that the apatite-like materials were formed with higher levels of crystallinity. The XRD data confirmed the apatite-like materials formed in polyurethane/CaCl_2_ composite nanofiber, which is in consistent with SEM data.

**Figure 5:**
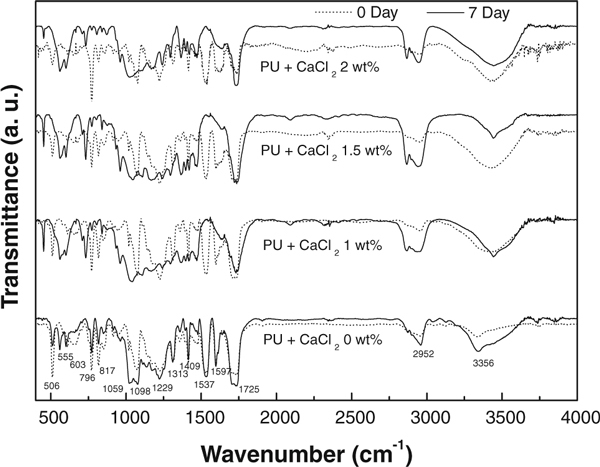
**FTIR spectra of the electrospun polyurethane nanofibers containing different concentration of CaCl_2_ salt before and after incubation in SBF solution**.

FTIR spectroscopy was performed to investigate the functional groups present in the polyurethane/CaCl_2_ composite nanofibers. Figure 5 shows the FTIR spectra of the polyurethane nanofibers with different concentrations of CaCl_2_ before and after incubation in SBF solution. These data clearly revealed the presence of the various vibrational modes corresponding to phosphates and hydroxyl groups. As shown in this figure, the characteristics transmittance bands of the phosphate ion, which appear from 600 to 1,500 cm^-1^, can be observed in the polyurethane/CaCl_2_ composite nanofibers. Within this range, the following main peaks at 817 cm^-1^ (ν_1_), 1,059 cm^-1^ (ν_2_) and 1,098 cm^-1^ (ν_3_), 555 and 603 cm^-1^ (ν_4_) were assigned to PO_4_^3-^. And the broad transmittance at 3,356 cm^-1^ was attributed to the–OH stretching. The intensity of–OH band was observed to be lower for the pristine polyurethane than those of the samples containing CaCl_2_ owing to the formation of apatite-like materials. In addition, the bands corresponding to CO_3_^2-^ was observed at the wavenumbers 817, 1,409 and 1,537 cm^-1^, owing to a small amount of CO_3_^2-^ ions present in the apatite structure [2,24,25]. Comparing the spectra of the two cases of 0-day and 7-day incubation in SBF solution, the intensity of the transmittance bands was increased with increasing CaCl_2_ concentration.

**Figure 6:**
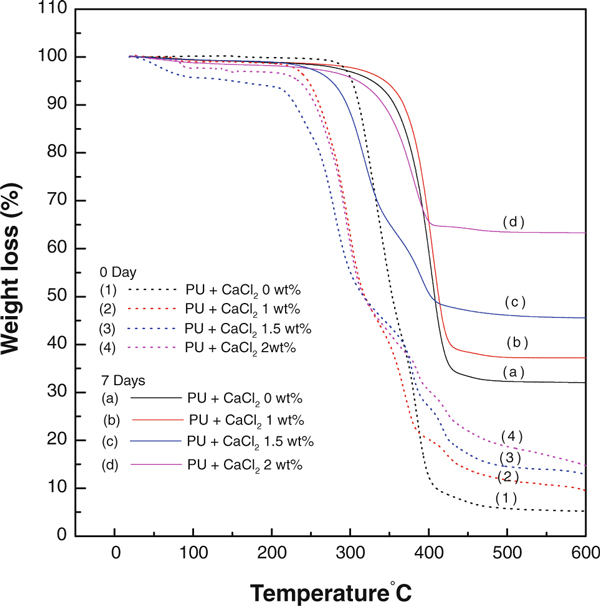
**TGA graphs of the electrospun polyurethane nanofibers containing different concentration of CaCl_2_ salt before and after incubation in SBF solution**.

TGA analyses were performed to confirm the formation of apatite-like materials in the polyurethane/CaCl_2_ composite nanofibers upon incubation in SBF solution. Figure 6 shows the TGA analyses of polyurethane containing CaCl_2_ salt for before and after incubation in SBF solution. The TGA results showed that the polyurethane nanofibers were decomposed in a single step. The onset decomposition temperature of polyurethane nanofibers was 425°C. As expected, the induction of biological apatite material over nanofibers surfaces would result in an increased residual weight during TGA analyses. It was observed that the polyurethane/CaCl_2_ composite nanofibers after incubation in SBF solution showed high residual weights than those without incubation. The residual weight dramatically increased with increasing CaCl_2_ concentration in the polyurethane nanofibers as shown in solid lines in Figure 6. This result clearly confirmed that the nucleation effect was increased with increasing CaCl_2_ concentration in polyurethane nanofibers. The weight loss dramatically increased for the sample with CaCl_2_ content of 2 wt% in which the CaCl_2_ acted as a heterogeneous nucleating agent. Similar kind of phenomenon was also recently reported on electrospun composite consisting of hydroxyapatite/β-tricalcium phosphate and poly(ε-caprolactone) as a potential scaffold for bone tissue engineering application [26]. The presence of CaCl_2_ in polyurethane nanofibers accelerates the formation of apatite aggregation during incubation in SBF solution. The TGA data are in good agreement with SEM results in which the apatite materials were formed on the nanofibers surfaces upon incubation in SBF solution.

**Figure 7:**
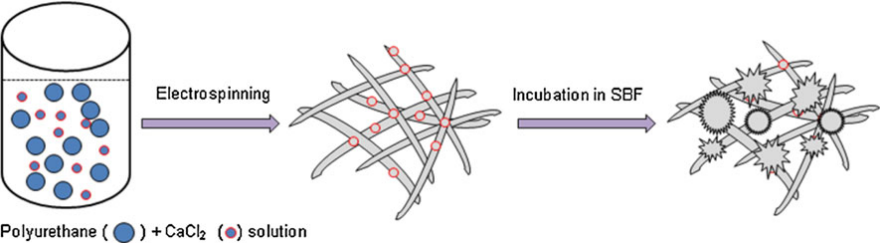
**Schematic illustration of hydroxyapatite mineralization on polyurethane/CaCl_2_ composite nanofibers mat**.

Figure 7 shows the schematic diagram illustrating the experimental process of the mineralization of hydroxyapatite on the polyurethane/CaCl_2_ composite nanofibers during immersion in SBF. The presence of calcium in polyurethane nanofibers accelerates the formation of apatite aggregation during incubation in the SBF solution. Development of the apatite-like materials on the polyurethane/CaCl_2_ composite nanofiber surface depends on the presence of nucleation sites and sufficient concentration of ionic species necessary to form the apatite. Relatively high specific surface area of the rugged calcium ions on the surfaces of the polyurethane/CaCl_2_ composite nanofibers provides the nucleation sites for apatite in SBF compared with pristine polyurethane nanofibers, results in more energetic nucleation sites. When a nucleus forms on the calcium ions, the contact area between nanofiber surface and nucleus is small enough such that a preferred orientation growth habit is active and initiates faster growth. Consequently, a large number of apatite nuclei are formed on the polyurethane/CaCl_2_ composite nanofibers surface. Then, the ions necessary for the growth can diffuse to the nucleation site from all directions and provide many nucleation sites resulting in spherical bunches of apatite-like material growth [see Figure 2a4]. Thus in the development of the biomimetic reaction on the polyurethane/CaCl_2_ composite nanofibers, the bone-like apatite crystals appear to be formed via intermediate products of calcium phosphate. The result demonstrated the fast nucleation of apatite-like materials with increasing concentration of CaCl_2_ in polyurethane composite nanofibers, which can be utilized for the guided bone regeneration membrane.

## Conclusions

Polyurethane nanofibers containing different concentrations of CaCl_2_ were successfully produced by electrospinning technique. These as-spun nanofibers exhibited a smooth surface and uniform diameters without any beads along their lengths. Addition of CaCl_2_ salt resulted in the formation of mesh-like ultrafine nanofibers in between the main fibers. The formation of apatite-like materials was spontaneously commenced, and their size was monotonically increased with increasing CaCl_2_ concentration after incubation in SBF solution. Good attachment and fast proliferation of apatite-like materials was observed in SEM analysis after incubation in SBF solution. The EDX and XRD data clearly confirmed the presence of apatite-like materials in the polyurethane/CaCl_2_ composite nanofibers. The results of FTIR and TGA analyses were also revealed the formation of plenty of apatite-like materials on the surfaces of polyurethane/CaCl_2_ composite nanofibers. Because of good attachment and formation behaviors, there is a potential to utilize polyurethane/CaCl_2_ composite nanofibers scaffold for the bone regeneration applications.
